# 2-Chloro-6-methyl­pyrimidin-4-amine

**DOI:** 10.1107/S1600536812047794

**Published:** 2012-11-28

**Authors:** Su-Lan Dong, Xiaochun Cheng

**Affiliations:** a College of Life Science and Chemical Engineering, Huaiyin Institute of Technology, Huaiyin 223003, Jiangsu, People’s Republic of China

## Abstract

In the crystal structure of the title compound, C_5_H_6_ClN_3_, mol­ecules are linked by pairs of N—H⋯N hydrogen bonds, forming inversion dimers. These dimers are linked *via* N—H⋯N hydrogen bonds, forming a two-dimensional network lying parallel to (100). Inversion-related mol­ecules are also linked *via* a slipped π–π inter­action, with a centroid–centroid distance of 3.5259 (11) Å, a normal separation of 3.4365 (7) Å and a slippage of 0.789 Å.

## Related literature

The title compound is an important organic inter­mediate which has been used to synthesise a drug that has shown promising activity against, for example, inflammatory bowel disease. For the synthetic procedure, see: Graceffa *et al.* (2010 ▶). For bond-length data, see: Allen *et al.* (1987 ▶).
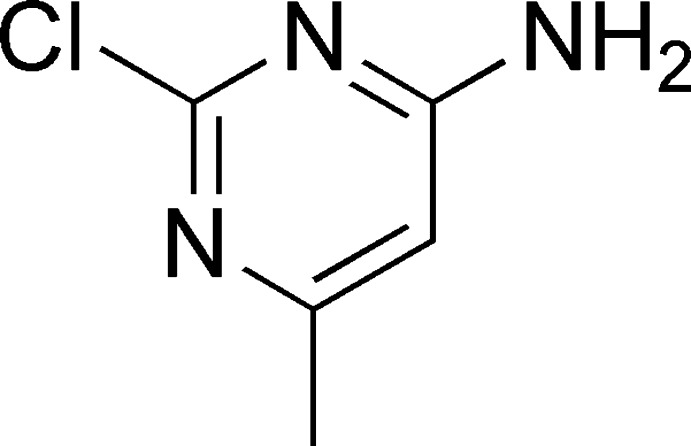


## Experimental

### 

#### Crystal data

C_5_H_6_ClN_3_
*M* *_r_* = 143.58
Monoclinic, 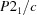

*a* = 7.1256 (8) Å
*b* = 7.8537 (8) Å
*c* = 13.0769 (15) Å
β = 115.678 (1)°
*V* = 659.54 (13) Å^3^
*Z* = 4
Mo *K*α radiation
μ = 0.48 mm^−1^
*T* = 296 K
0.14 × 0.12 × 0.12 mm

#### Data collection

Enraf–Nonius CAD-4 diffractometer
Absorption correction: ψ scan (North *et al.*, 1968 ▶) *T*_min_ = 0.935, *T*_max_ = 0.944
5910 measured reflections
1157 independent reflections
1103 reflections with *I* > 2σ(*I*)
*R*_int_ = 0.077
3 standard reflections every 200 reflections intensity decay: 1%

#### Refinement

*R*[*F* ^2^ > 2σ(*F* ^2^)] = 0.057
*wR*(*F* ^2^) = 0.143
*S* = 1.17
1157 reflections
83 parameters
H-atom parameters constrained
Δρ_max_ = 0.36 e Å^−3^
Δρ_min_ = −0.76 e Å^−3^

### 

Data collection: *CAD-4 Software* (Enraf–Nonius, 1985 ▶); cell refinement: *CAD-4 Software*; data reduction: *XCAD4* (Harms & Wocadlo,1995 ▶); program(s) used to solve structure: *SHELXS97* (Sheldrick, 2008 ▶); program(s) used to refine structure: *SHELXL97* (Sheldrick, 2008 ▶); molecular graphics: *SHELXTL* (Sheldrick, 2008 ▶); software used to prepare material for publication: *SHELXTL*.

## Supplementary Material

Crystal structure: contains datablock(s) I, global. DOI: 10.1107/S1600536812047794/su2530sup1.cif

Structure factors: contains datablock(s) I. DOI: 10.1107/S1600536812047794/su2530Isup2.hkl

Supplementary material file. DOI: 10.1107/S1600536812047794/su2530Isup3.cml

Additional supplementary materials:  crystallographic information; 3D view; checkCIF report

## Figures and Tables

**Table 1:** Hydrogen-bond geometry (Å, °)

*D*—H⋯*A*	*D*—H	H⋯*A*	*D*⋯*A*	*D*—H⋯*A*
N3—H3*A*⋯N2^i^	0.86	2.24	3.090 (3)	170
N3—H3*B*⋯N1^ii^	0.86	2.26	3.045 (2)	152

Symmetry codes: (i) 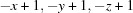; (ii) 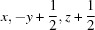.

**Fig. 1.**
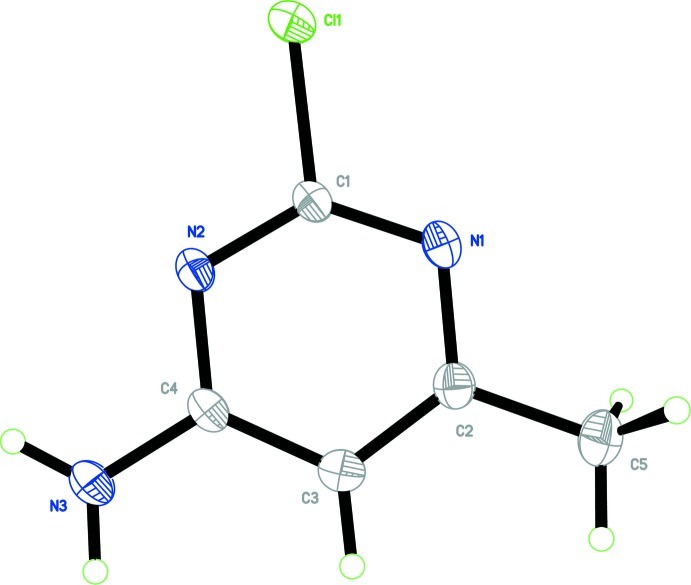
The molecular structure of the title compound, with the atom numbering. Displacement ellipsoids are drawn at the 50% probability level.

**Fig. 2.**
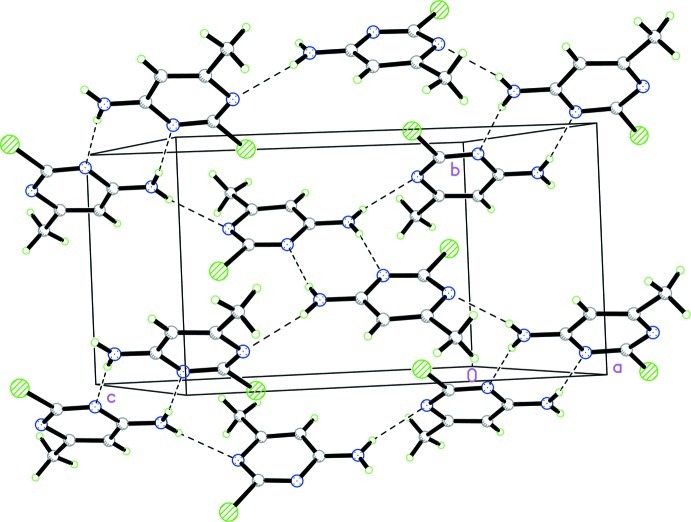
A view of the crystal packing of the title compound. The N—H···N hydrogen bonds are shown as dashed lines (see Table 1 for details).

## supplementary crystallographic information

### Comment

The title compound is an important organic intermediate which has been used to synthesis drugs which have show promising activity against diseases, such as asthma, inflammatory bowel disease, and Crohn's disease (Graceffa *et al.*, 2010). Herein we report on the crystal structure of the title compound.

The molecular structure of the title molecule is shown in Fig. 1. The bond lengths (Allen *et al.*, 1987) and angles are within normal ranges.

In the crystal, molecules are linked by pairs of N-H···N hydrogen bonds forming inversion dimers. These dimers are linked via N-H···N hydrogen bonds forming a two-dimensional network lying parallel to plane (100). See Table 1 and Fig. 2 for details. Inversion related molecules are also linked via a slipped π-π interaction with a centroid-to-centroid distance of 3.5259 (11) Å ; a normal separation of 3.4365 (7) Å; slippage of 0.789 Å (Cg1···Cg1^i^ where Cg1 is the N1/C1/N2/C4/C3/C2 ring; symmetry code: (i) -x+2, -y+1, -z+1).

### Experimental

The title compound was prepared by the method reported in the literature (Graceffa *et al.*, 2010). A solution of 2-chloro-4-methyl-6-nitropyrimidine (5 g, 15.77 mmol) in dichloromethane (50 ml) was added slowly to a solution of iron powder and hydrochloric acid (10 g, 178 mmol). After being stirred for 6 h at room temperature, the solution was filtered and the organic phase was evaporated on a rotary evaporator and gave the title compound. Block-like colourless crystals were obtained by slow evaporation of a solution of the title compound (0.5 g, 3.5 mmol) in ethanol (25 ml), at room temperature after ca. 7 d.

### Refinement

All H atoms were positioned geometrically and refined using a riding model: N-H = 0.86 Å, C—H = 0.93 and 0.96 Å for aromatic and CH_3_ H atoms, respectively, with *U*_iso_(H) = k × *U*_eq_(N,C), where k = 1.5 for CH_3_ H atoms and = 1.2 for other H atoms.

### Crystal data

**Table:**

C_5_H_6_ClN_3_	*F*(000) = 296
*M**_r_* = 143.58	*D*_x_ = 1.446 Mg m^−^^3^
Monoclinic, *P*2_1_/*c*	Mo *K*α radiation, λ = 0.71073 Å
Hall symbol: -P 2ybc	Cell parameters from 5910 reflections
*a* = 7.1256 (8) Å	θ = 3.2–25.0°
*b* = 7.8537 (8) Å	µ = 0.48 mm^−^^1^
*c* = 13.0769 (15) Å	*T* = 296 K
β = 115.678 (1)°	Block, colourless
*V* = 659.54 (13) Å^3^	0.14 × 0.12 × 0.12 mm
*Z* = 4	

### Data collection

**Table:**

Enraf–Nonius CAD-4 diffractometer	1103 reflections with *I* > 2σ(*I*)
Radiation source: fine-focus sealed tube	*R*_int_ = 0.077
Graphite monochromator	θ_max_ = 25.0°, θ_min_ = 3.2°
ω/2θ scans	*h* = −8→8
Absorption correction: ψ scan (North *et al.*, 1968)	*k* = −8→9
*T*_min_ = 0.935, *T*_max_ = 0.944	*l* = −15→15
5910 measured reflections	3 standard reflections every 200 reflections
1157 independent reflections	intensity decay: 1%

### Refinement

**Table:**

Refinement on *F*^2^	Primary atom site location: structure-invariant direct methods
Least-squares matrix: full	Secondary atom site location: difference Fourier map
*R*[*F*^2^ > 2σ(*F*^2^)] = 0.057	Hydrogen site location: inferred from neighbouring sites
*wR*(*F*^2^) = 0.143	H-atom parameters constrained
*S* = 1.17	*w* = 1/[σ^2^(*F*_o_^2^) + (0.0881*P*)^2^ + 0.2103*P*] where *P* = (*F*_o_^2^ + 2*F*_c_^2^)/3
1157 reflections	(Δ/σ)_max_ = 0.004
83 parameters	Δρ_max_ = 0.36 e Å^−^^3^
0 restraints	Δρ_min_ = −0.76 e Å^−^^3^

### Special details

**Table:**

Geometry. All e.s.d.'s (except the e.s.d. in the dihedral angle between two l.s. planes) are estimated using the full covariance matrix. The cell e.s.d.'s are taken into account individually in the estimation of e.s.d.'s in distances, angles and torsion angles; correlations between e.s.d.'s in cell parameters are only used when they are defined by crystal symmetry. An approximate (isotropic) treatment of cell e.s.d.'s is used for estimating e.s.d.'s involving l.s. planes.
Refinement. Refinement of *F*^2^ against ALL reflections. The weighted *R*-factor *wR* and goodness of fit *S* are based on *F*^2^, conventional *R*-factors *R* are based on *F*, with *F* set to zero for negative *F*^2^. The threshold expression of *F*^2^ > σ(*F*^2^) is used only for calculating *R*-factors(gt) *etc*. and is not relevant to the choice of reflections for refinement. *R*-factors based on *F*^2^ are statistically about twice as large as those based on *F*, and *R*- factors based on ALL data will be even larger.

### Fractional atomic coordinates and isotropic or equivalent isotropic displacement parameters (Å^2^)

**Table:**

	*x*	*y*	*z*	*U*_iso_*/*U*_eq_	
C1	0.6937 (3)	0.4212 (2)	0.33622 (15)	0.0279 (4)	
C2	0.9835 (3)	0.2642 (2)	0.42236 (17)	0.0304 (5)	
C3	0.9496 (3)	0.2568 (2)	0.51731 (17)	0.0336 (5)	
H3	1.0404	0.1980	0.5814	0.040*	
C4	0.7729 (3)	0.3408 (2)	0.51565 (15)	0.0286 (4)	
C5	1.1670 (3)	0.1823 (3)	0.4147 (2)	0.0463 (6)	
H5A	1.2551	0.1315	0.4863	0.069*	
H5B	1.2442	0.2669	0.3958	0.069*	
H5C	1.1193	0.0961	0.3569	0.069*	
Cl1	0.51828 (9)	0.52705 (8)	0.21459 (4)	0.0494 (3)	
N1	0.8510 (2)	0.34779 (19)	0.32654 (13)	0.0305 (4)	
N2	0.6439 (2)	0.42741 (19)	0.42172 (13)	0.0288 (4)	
N3	0.7236 (3)	0.3407 (3)	0.60284 (15)	0.0416 (5)	
H3A	0.6144	0.3939	0.5978	0.050*	
H3B	0.8009	0.2874	0.6642	0.050*	

### Atomic displacement parameters (Å^2^)

**Table:**

	*U*^11^	*U*^22^	*U*^33^	*U*^12^	*U*^13^	*U*^23^
C1	0.0323 (9)	0.0294 (9)	0.0252 (9)	−0.0011 (7)	0.0154 (7)	−0.0003 (7)
C2	0.0321 (9)	0.0291 (9)	0.0321 (11)	−0.0023 (7)	0.0158 (7)	−0.0051 (7)
C3	0.0348 (10)	0.0346 (10)	0.0300 (10)	0.0008 (7)	0.0126 (8)	0.0023 (7)
C4	0.0340 (9)	0.0300 (9)	0.0250 (9)	−0.0042 (7)	0.0159 (7)	−0.0013 (7)
C5	0.0422 (11)	0.0513 (13)	0.0525 (14)	0.0096 (10)	0.0271 (10)	−0.0002 (10)
Cl1	0.0559 (5)	0.0652 (5)	0.0326 (4)	0.0224 (3)	0.0245 (3)	0.0176 (2)
N1	0.0348 (8)	0.0331 (8)	0.0289 (9)	−0.0021 (6)	0.0187 (7)	−0.0044 (6)
N2	0.0332 (8)	0.0315 (8)	0.0267 (8)	−0.0006 (6)	0.0177 (7)	−0.0003 (6)
N3	0.0466 (10)	0.0571 (11)	0.0284 (9)	0.0088 (8)	0.0232 (8)	0.0095 (8)

### Geometric parameters (Å, º)

**Table:**

C1—N2	1.313 (3)	C4—N3	1.331 (3)
C1—N1	1.315 (2)	C4—N2	1.356 (2)
C1—Cl1	1.7494 (18)	C5—H5A	0.9600
C2—N1	1.366 (3)	C5—H5B	0.9600
C2—C3	1.365 (3)	C5—H5C	0.9600
C2—C5	1.499 (3)	N3—H3A	0.8600
C3—C4	1.413 (3)	N3—H3B	0.8600
C3—H3	0.9300		
			
N2—C1—N1	130.94 (17)	C2—C5—H5A	109.5
N2—C1—Cl1	113.97 (13)	C2—C5—H5B	109.5
N1—C1—Cl1	115.09 (14)	H5A—C5—H5B	109.5
N1—C2—C3	122.03 (16)	C2—C5—H5C	109.5
N1—C2—C5	114.88 (18)	H5A—C5—H5C	109.5
C3—C2—C5	123.09 (19)	H5B—C5—H5C	109.5
C2—C3—C4	118.28 (17)	C1—N1—C2	113.67 (16)
C2—C3—H3	120.9	C1—N2—C4	115.17 (16)
C4—C3—H3	120.9	C4—N3—H3A	120.0
N3—C4—N2	116.70 (17)	C4—N3—H3B	120.0
N3—C4—C3	123.43 (18)	H3A—N3—H3B	120.0
N2—C4—C3	119.88 (17)		

### Hydrogen-bond geometry (Å, º)

**Table:**

*D*—H···*A*	*D*—H	H···*A*	*D*···*A*	*D*—H···*A*
N3—H3*A*···N2^i^	0.86	2.24	3.090 (3)	170
N3—H3*B*···N1^ii^	0.86	2.26	3.045 (2)	152

Symmetry codes: (i) −*x*+1, −*y*+1, −*z*+1; (ii) *x*, −*y*+1/2, *z*+1/2.
